# Genome-Wide Association Studies of Anthracnose and Angular Leaf Spot Resistance in Common Bean (*Phaseolus vulgaris* L.)

**DOI:** 10.1371/journal.pone.0150506

**Published:** 2016-03-01

**Authors:** Juliana Morini Küpper Cardoso Perseguini, Paula Rodrigues Oblessuc, João Ricardo Bachega Feijó Rosa, Kleber Alves Gomes, Alisson Fernando Chiorato, Sérgio Augusto Morais Carbonell, Antonio Augusto Franco Garcia, Rosana Pereira Vianello, Luciana Lasry Benchimol-Reis

**Affiliations:** 1 Universidade Tecnológica Federal do Paraná (UTFPR), Dois Vizinhos, Paraná, Brasil; 2 Centro de Recursos Genéticos Vegetais, Instituto Agronômico de Campinas (IAC), Campinas, São Paulo, Brazil; 3 Laboratório Nacional de Biociências (LNBio), Campinas, São Paulo, Brazil; 4 Departamento de Genética, Escola Superior de Agricultura Luiz de Queiroz/Universidade de São Paulo (ESALQ/USP), Piracicaba, São Paulo, Brazil; 5 Centro de Grãos e Fibras, Instituto Agronômico de Campinas (IAC), Campinas, São Paulo, Brazil; 6 Empresa Brasileira de Pesquisa Agropecuária, Centro Nacional de Pesquisa de Arroz e Feijão, Goiania, Goiás, Brazil; National Institute of Plant Genome Research (NIPGR), INDIA

## Abstract

The common bean (*Phaseolus vulgaris* L.) is the world’s most important legume for human consumption. Anthracnose (ANT; *Colletotrichum lindemuthianum*) and angular leaf spot (ALS; *Pseudocercospora griseola*) are complex diseases that cause major yield losses in common bean. Depending on the cultivar and environmental conditions, anthracnose and angular leaf spot infections can reduce crop yield drastically. This study aimed to estimate linkage disequilibrium levels and identify quantitative resistance loci (QRL) controlling resistance to both ANT and ALS diseases of 180 accessions of common bean using genome-wide association analysis. A randomized complete block design with four replicates was performed for the ANT and ALS experiments, with four plants per genotype in each replicate. Association mapping analyses were performed for ANT and ALS using a mixed linear model approach implemented in TASSEL. A total of 17 and 11 significant statistically associations involving SSRs were detected for ANT and ALS resistance loci, respectively. Using SNPs, 21 and 17 significant statistically associations were obtained for ANT and angular ALS, respectively, providing more associations with this marker. The SSR-IAC167 and PvM95 markers, both located on chromosome Pv03, and the SNP scaffold00021_89379, were associated with both diseases. The other markers were distributed across the entire common bean genome, with chromosomes Pv03 and Pv08 showing the greatest number of loci associated with ANT resistance. The chromosome Pv04 was the most saturated one, with six markers associated with ALS resistance. The telomeric region of this chromosome showed four markers located between approximately 2.5 Mb and 4.4 Mb. Our results demonstrate the great potential of genome-wide association studies to identify QRLs related to ANT and ALS in common bean. The results indicate a quantitative and complex inheritance pattern for both diseases in common bean. Our findings will contribute to more effective screening of elite germplasm to find resistance alleles for marker-assisted selection in breeding programs.

## Introduction

Common bean (*Phaseolus vulgaris* L.) is an annual legume crop with a relatively small genome of 473 Mb [1]. It is the most important grain legume for direct human consumption [2,3]. Dry beans provide a major source of quality protein, which is high in lysine and therefore complements most cereals. In addition, beans are high in carbohydrates, fiber, and minerals (calcium, potassium, phosphorus iron, zinc and magnesium) [4].

Anthracnose (ANT) and Angular Leaf Spot (ALS) are two important diseases that have the greatest impact on crop yield reduction. Yield losses caused by the ANT pathogen are extremely high in common bean, reaching up to 100% [5]. Anthracnose is caused by the specialized hemibiotrophic fungus *Colletotrichum lindemuthianum* (Sacc. and Magnus), which co-evolved with common bean into Andean and Mesoamerican races [6,7]. ALS leads to crop losses of up to 80% and is found in more than 60 countries worldwide [8,9]. This disease is caused by the hemibiotrophic fungus *Pseudocercospora griseola* (Sacc.) Crous & Braun (sin. *Phaeoisariopsis griseola* (Sacc.) Ferraris) [10], and can be identified by angular necrotic spots on plant leaves and pods [11].

Bi-parental populations used for QRL mapping accumulate a limited number of recombination events, typically leading to low mapping resolution or poor estimation of marker effects [12,13]. In this case, recombination has not had sufficient time to shuffle the genome into small fragments, and quantitative trait loci (QTL) are generally localized to large chromosomal regions of 10 to 20 centiMorgan [14].

Genome-wide association studies (GWAS) or association mapping (AM) and linkage disequilibrium (LD) offer high resolution through historical recombination accumulated in natural populations and collections of landraces, breeding materials, and varieties [15]. By exploiting broader genetic diversity, GWAS offers several advantages over linkage mapping, such as mapping resolution, allele number, time saved in establishing marker-trait associations, and application in breeding programs [16]. The strength of the correlation between two markers is a function of the distance between them: the closer two markers are, the stronger the LD. The resolution with which a QRL can be mapped is a function of how quickly LD decays over distance. Selfing reduces opportunities for recombination; thus, in self-pollinating species such as rice (*Oryza Sativa*), LD may extend to 100 Kb or more [17]. In general, high LD is expected between tightly linked loci, since the mutation should have eliminated LD between loci that are not in close proximity to one another [18,19]. In common bean, little information is available on the extent of LD [20,21,22].

The Common Bean Germplasm Bank of the Agronomic Institute (IAC, Campinas, S.P. Brazil) holds more than 1800 accessions representing the two principal centers of origin (Andean and Mesoamerican) of the species as well as ecotypes from different South American countries and a large number of lines from both Brazilian and international genetic breeding programs, most of which were obtained by germplasm interchange [23]. Some factors should be considered for the improvement association mapping, one of which is the selection of a group of individuals from the original germplasm collection with coverage of a high level of genetic diversity [24]. In a previous study [25], evaluated the diversity level and genetic organization present among 500 accessions from the Common Bean Germplasm Bank of the IAC and selected 180 accessions that represented the variability of the entire collection in order to use this panel for association mapping studies.

Some points in association mapping there are to consider: (1) selection of a germplasm collection with high level of genetic diversity; (2) recording or measuring the phenotypic characteristics of the selected population groups; (3) genotyping the individuals with available molecular markers; (4) quantification of the LD extent of the genome of a chosen population using molecular markers; (5) assessment of the level of genetic differentiation among groups within the sampled individuals and the coefficient of relatedness between pairs of individuals within a sample; and (6) taking the information gained through the quantification of LD and population structure into account for the correlation of phenotypic and genotypic/haplotypic data with the application of an appropriate statistical approach that reveals “marker tags” positioned within close proximity of the targeted trait of interest [26].

Several groups have performed AM studies for common bean [27,28,29], but none have focused on ANT and ALS resistance. Based on several linkage analysis studies, we have reported previously important resistance candidate genes in common bean [30,31,32,33]. However, to complement our initial findings and further understand the ANT and ALS plant disease interactions in common bean, the aims of this study were to (i) estimate the linkage disequilibrium (LD) in common bean; and (ii) identify QRLs associated with resistance to ANT and ALS using SSR and SNP data.

## Materials and Methods

### Plant material and DNA extraction

One hundred and eighty accessions previously identified and evaluated [25] from the common bean germplasm bank of the Agronomic Institute (IAC, Campinas, São Paulo, Brazil) were used in this work, with 24 genotypes are of Andean origin and 156 are of Mesoamerican origin. These genotypes displayed variance in many agronomic traits related to resistance to major common bean diseases (anthracnose, angular leaf spot, rust, fusarium wilt, bacterial blight, and gold mosaic virus), drought tolerance, as well as differences in grain size and tegument color. In summary, among the 180 accessions [25], 75 were chosen due to their great economic importance to the Brazilian common bean breeding program, as they include commercial varieties with carioca tegument, widely cultivated in at the state of São Paulo (Brazil), under the leadership of the Agronomic Institute (IAC). Total genomic DNA (gDNA) was extracted from lyophilized young leaf tissues using the CTAB method.

### Simple sequence repeat (SSR) marker analysis

A total of 103 microsatellites (SSRs) were amplified. From these, 45 were EST-SSRs (EST—Expressed sequence tag) [34], while the others were genomic-SSRs [35]. Polymerase chain reaction (PCR) amplifications were performed in a 25 μL final volume containing 50 ng DNA template, 1× buffer, 0.2 μM forward primer, 0.2 μM reverse primer, 100 μM dNTP, 2.0 mM MgCl_2_, 10 mM Tris-HCl (pH 8,0), 50 mM KCl, and 0.5 U of *Taq* DNA polymerase. The following conditions were used for amplification: 1 min at 94°C, 30 cycles of 1 min at 94°C, 1 min at the specific annealing temperature for each SSR, and 1 min at 72°C, with a final extension of 5 min at 72°C. The PCR products were checked on a 3% agarose gel and separated using 6% silver-stained polyacrylamide.

Allele sizes were scored in base pairs (bp) by visual comparison with a 10-bp DNA ladder (Invitrogen), and the value was converted to gene and genotypic frequencies. After the binary allele scoring (1 or 0) was completed, genotyping was performed using the allele number in decreasing order; that is, alleles with greater size received the highest numbers. In the case of diploids such as common bean, homozygous bands with heterozygous genotypes were scored twice.

### Single-nucleotide polymorphism (SNP) marker analysis

SNP genotyping was conducted using the technology Vera Code^®^ BeadXpress platform (Illumina) at the Biotechnology Laboratory of Embrapa (Goiania, GO, Brazil). A set of 384 SNP markers, validated by a previously identified Prelim file (https://icom.illumina.com/Custom/UploadOpaPrelim/) for *Phaseolus vulgaris* [36], a derivative of polymorphisms between strains BAT477 and Pérola of Mesoamerican origins, was selected to compose the Oligo Pool Assay (OPA) SNP marker panel. Three oligonucleotides were used for each of the variants of the same SNP and the third specific-locus binding to the 3′ region of the DNA fragment containing the target SNP, generating a unique allele-specific fragment. Subsequently, this fragment was amplified using *Taq* DNA polymerase enzyme Titanium (Clontech^®^) and complementary primers labeled with Cy3 and Cy5 fluorophores. Genotyping was performed using Genome Studio software version 1.8.4 (Illumina, EUA) using Call Rate values ranging from 0.80 to 0.90 and GenTrain ≥ 0.26 for SNP grouping. Automated analyses were performed to cluster the SNP alleles of each line, based on signal intensities of Cy3 and Cy5 fluorophores. Groups were adjusted manually by determining the best clusters based on parental profiles.

### Linkage disequilibrium (LD) analysis

Fisher’s exact tests [37] were performed for each possible pair of markers from the 103 SSRs. To avoid false positives due to multiple tests (i.e., markers in linkage equilibrium or non-causative associations), Bonferroni [38] and False Discovery Rate (FDR) [39] corrections were applied. FDR was applied in addition to Bonferroni because the latter is a very conservative method for type I error control. Furthermore, to determine the suitable marker density resolution for association mapping, we also analyzed the extent of LD against the genetic distance considering only Fisher’s exact test results between the mapped and linked SSRs, based on two previously published genetic linkage maps for common bean [35,40]. All the analyses were performed using R software (R Development Core Team 2014).

For 384 SNPs, pairwise tests for LD levels were performed using r^2^ [41], which was proposed in the context of biallelic loci. Initially, LD was estimated for each chromosome separately, and then a heat map for each one was generated to view LD patterns. Subsequently, the LD obtained for all associations involving linked SNPs from the different common bean chromosomes was plotted against the genetic distance. With these associations, it was possible to adjust a non-linear model for LD decay. All the analyses were performed using the package *synbreed* [42] available in R software (R Development Core Team 2014).

### Phenotypic analyses of anthracnose and angular leaf spot

For the ANT phenotype the seeds of 180 common bean accessions were germinated on germination paper in a growth chamber at 25°C with a 12-hours photoperiod for 3 days. Four seedlings per accession were transplanted to boxes containing autoclaved vermiculite (Plantmax^®^) as substrate, constituting an experimental plot. Four different accessions were grown per box. The experimental design was carried out in randomized complete blocks with four replicates. Plants were inoculated 7 days after transplanting, where each experiment consisted in a single race of *C*. *lindemuthianum* inoculation (race 04). The experimental design was carried out in randomized complete blocks with four replicates.

Monosporic cultures of *C*. *lindemuthianum* were grown on PDA media (200 g L^–1^ potato, 30 g L^–1^ dextrose and 30 g L^–1^ agar), and conidia were collected in water suspension using a glass spreader. Plants were sprayed with spore suspension (10^6^ spores mL^–1^) using a DeVilBiss apparatus (Fanem). Immediately after inoculation, plants were kept for 48 hours under 95–100% relative humidity at 23°C and a 12-hour photoperiod. Disease severity was evaluated 7 to 10 days post-inoculation using a diagrammatic scale proposed by Pastor-Corrales et al., in which 1–3 denotes resistant, 4–6 intermediate, and 7–9 susceptible [6].

For the ALS phenotype the seeds were sown in plastic boxes (29.5 cm × 46.5 cm × 12.5 cm) containing Dystrophic Red Latosol type soil, fertilized with NPK 04-14-08 (400 kg/ha), each with four accessions sown in rows at a distance of approximately 4 cm, containing four plants per row. The experimental design was carried out in randomized complete blocks with four replicates. Plants were inoculated when they reached the V3 development stage (first expanded trifoliate), 2 to 3 weeks after planting, by spraying both leaf surfaces with a 10^4^ conidia/mL suspension prepared from *P*. *griseola* monosporic colonies grown in V8 medium [43]. The isolate used (14259–2) was classified into the 0–39 race based on the response of the differential cultivars according to Pastor-Corrales et al. [6].

After inoculation, the accessions were held for 48 hours at a temperature between 22°C to 24°C, relative humidity between 95–100%, and a photoperiod of 12 hours [43]. After this period, plants were transferred to the greenhouse. The evaluation of severity was made 17 days after inoculation.

The race 04 of *C*. *lindemuthianum* was used in this study because it previously detected ANT QTLs in greenhouse conditions [32], and the race 0–39 of *P*. *griseola* was also previously associated to ALS QTLs [30,31].

The phenotypic data obtained for each disease (ANT and ALS), based on the 9-point scale of severity, were analyzed according to the following statistical model:

$$Y_{ijk}=\mu +b_{i}+g_{j}+r_{k\left(ij\right)}+\varepsilon_{ij}.$$

where *y*_*ijk*_ corresponds to the level of ANT or ALS severity; *μ* μis the general average; *b*_*i*_ is the fixed effect of the block *i*; *g*_*j*_ is the random effect associated with accession *j*; *r*_*k(ij)*_ is the random effect associated with the replicate *k* nested within genotype *j* and block *i*; and *ɛ*_*ij*_ is the random residual term. The analyses were performed using the *nlme* package, available in R software (R Development Core Team 2014).

The presence of heterogeneous genetic variances and genetic covariances and correlations between the observations were investigated in the phenotypic analyses. We tested different mixed models for the genetic-effects matrix considering the interaction between the genotypes and the blocks or replicates, using the Akaike information criterion (AIC) [44] and Bayesian information criterion (BIC) [45] to compare and select the best models. The predicted means of the genotypes were obtained from the most likely models for ANT and ALS and used for association mapping analyses.

### Genome mapping and functional annotation

Molecular markers associated with ANT and ALS resistance were aligned to the common bean genome [1] using the native nucleotide basic local alignment search tool (BLASTn) and default algorithm parameters (threshold E-value < 1 × 10^−10^) from Phytozome version 1.0 (http://www.phytozome.net/). Putative candidate genes involved in the resistance of common bean to these diseases were identified from the genome localization of the markers. GO annotations for putative functions of the genes were obtained by comparison to GeneOntology (GO, http://www.geneontology.org/) using Blast2GO program [46]. The annotations were separated for each marker and the corresponding disease. The KEGG pathway annotations were obtained by aligning sequences to the KEGG database comparing to the protein sequences of Kyoto Encyclopedia of Genes and Genomes (KEGG, http://www.genome.jp/) [47]. To predict gene structure and marker locations into the gene, these markers were alignedwith phytozome annotated genomic sequence (http://www.phytozome.net/).

### Association mapping analyses

Marker’s sequences available on NCBI (http://www.ncbi.nlm.nih.gov/) or PhaseolusGenes (http://phaseolusgenes.bioinformatics.ucdavis.edu/) databases were used to localize the SSRs and SNPs in the *P*. *vulgaris* chromosomes using the native Phytozome’s BLAST and default algorithm parameters (http://www.phytozome.net/). The criteria used to assign putative chromosomes to the markers included E-values ≤ 1 × 10^−15^ and a minimum identity of 70% between query and database sequences.

Association mapping analyses were carried out using TASSEL version 2.01 software for SSRs and TASSEL version 5.0 for SNPs (http://www.maizegenetics.net/index.php?option=com_content&task=view&id=89&Itemid=119).

Mixed linear model (MLM) analyses were performed considering the estimated kinship matrix (K), calculated using SPAGeDi software [48], as random effects and the population structure matrix (Q), previously inferred by STRUCTURE software [49] in Perseguini et al. using SSRs, as part of the fixed effects [25]. The population structure consisted of a Q matrix that describes the percent subpopulation parentage for each line in the analysis. Cluster probabilities of K = 4 for SSRs and K = 3 for SNPs (results not shown) were used for the analyses.

## Results

### Analyses of linkage disequilibrium using SSRs and SNPs

**Fig 1:**
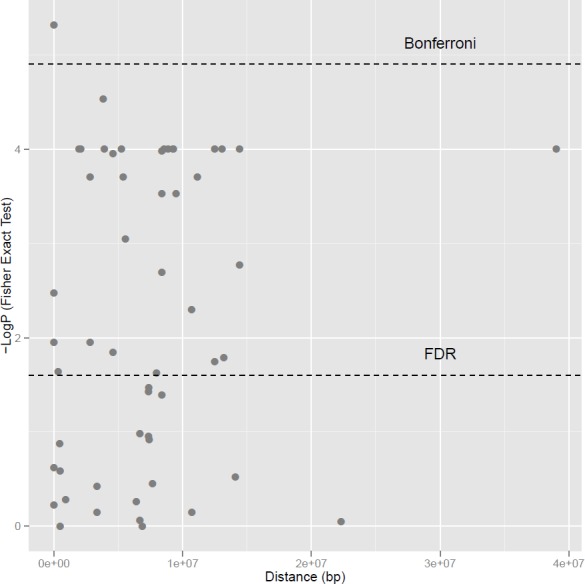
Linkage disequilibrium (r^2^) due to the genetic distance in bp, with SSRs markers. Thresholds corresponding to Bonferroni corrections and FDR are shown on a logarithmic scale with significant associations (in LD) and non-significant vectors above and below, respectively.

The analysis of LD using SSRs is showed in Fig 1. Based on the Bonferroni threshold (p-value: 1.24 x 10^−5^; -LogP: 4.904) used to control type I error, it was observed that non-random associations extended up to 100 cM for the common bean genome and then declined rapidly from that distance. Using the FDR threshold (p-value: 2.48 x 10^−2^; -LogP: 1.606), which is a less conservative procedure to control multiple tests, LD extended up to more than 300 cM. Thus, despite the fact that few associations in LD were obtained and plotted above 100 cM with Bonferroni correction, a higher and more extensive LD was observed for the common bean genome using FDR, as can be expected for selfing species such as beans.

**Fig 2:**
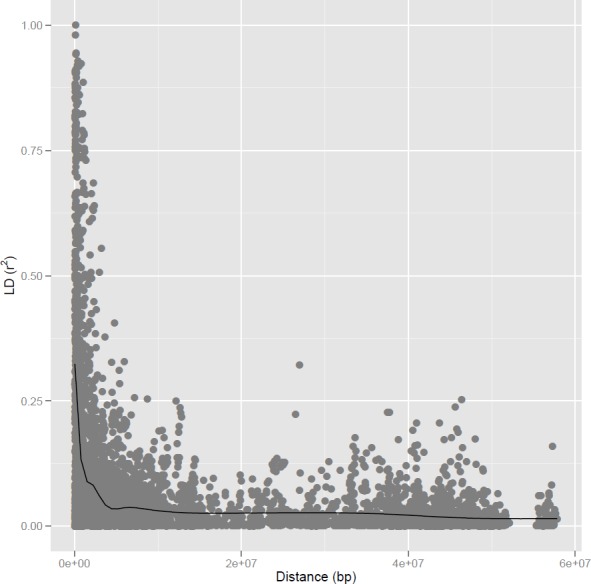
Allele pair linkage disequilibrium (r^2^) across the entire common bean genome for the full group of genotypes, due to the genetic distance in bp, with SNP markers.

From the 369 available SNPs, 331 could be used to calculate pairwise LD along the common bean genome. Using SNP markers, it was possible to detect a rapid and unexpected decay in LD throughout the genome (Fig 2), despite having a high and expected LD (higher values of *r*^*2*^) in various chromosomal regions (S1 Fig). This pattern suggests extensive LD blocks, particularly on chromosomes 3, 8, and 11. In general, the common bean LD extended to several megabases, as can be observed in Fig 2.

The extent of LD decay is usually affected by population structure. Assessing genome-wide LD patterns in beans is important not only for GWAS but also to shed light on historical effects of intensive directional selection due to domestication for vegetable use, compared with materials closer to the ancestral species. Examples have been reported between the *indica* and *japonica* groups of rice [50], wild and domesticated grape [51], landrace and modern varieties of wheat [52], and in asparagus bean (*Vigna unguiculata* ssp. *sesquipedialis* [53].

### Phenotypic analyses of anthracnose and angular leaf spot

A mixed model approach was used to analyze phenotypic data from ANT and ALS experiments. To investigate different structures of variances and covariances for the genetic effects, we considered all the accessions from the core collection as a random effect into the model (Table 1). Grouping for block effects or replicates within the blocks, we found that our data fitted better to complex mixed models. For both ANT and ALS, the diagonal (DIAG) model explained satisfactorily the observed genetic variation, based on the lower values of AIC and BIC criteria. This model considers the presence of heterogeneous genetic variances and absence of genetic covariances and correlations between observations for each level of the accessions.

**Table 1:** Different structures of variances and covariances for the genetic-effects matrix related to the blocks (*G*^Block^) and replicates within the genotypes and blocks (*G*^Rep^). Blocks and replicates are both used as grouping factor for each level of the random genotypes.

Matrix	Model	Parameter Number	ALS		ANT	
			AIC	BIC	AIC	BIC
$G=G^{\mathrm{Block}}$	ID	18	7,487.418	7,585.160	10,585.690	10,688.410
	DIAG	21	7,475.074	7,589.106	10,335.400	10,455.230
	CS	19	7,488.833	7,592.005	10,445.970	10,554.380
	UNST	27	NC	NC	NC	NC
$G=G^{\mathrm{Rep}}$	ID	18	7,474.923	7,572.665	10,331.630	10,434.340
	DIAG	34	7,318.306	7,502.930	10,313.830	NC
	CS	19	7,476.923	7,580.096	10,331.400	10,439.810
	UNST	–	NC	NC	NC	NC

G: random genetic-effects matrix; ID: Identity; DIAG: Diagonal; CS: Compound Symmetry; UNST: Unstructured; NC: No Convergence. The selected model for both ALS and ANT diseases is indicated by the value in bold, which correspond to AIC and BIC criteria for the first and only AIC for the latter.

### Association mapping using SSRs and SNPs

Of the 103 SSRs used in this study, 17 (16.5%) were associated with ANT (race 4) disease, of which three, PvM153 (Pv02), SSR-IAC254 (Pv08), and PvM98 (Pv11), were the most significant *(P* ≤ 0.001; Table 2). Statistically significant associations involving SSRs were observed for ANT in seven out of eleven chromosomes of the common bean (Table 2). For SNP markers, a total of 21 markers (6.3%) were associated with ANT disease (Table 2; S2 Fig). The SNP scaffold00024_916410 (Pv01), scaffold00060_874577 (Pv04), scaffold00021_89379 (Pv07), and sacaffold00034_860044 (Pv08) showed significant (*P* ≤ 0.001) associations with the trait. The SNP scaffold00021_89379 showed the most significant association to ANT, explaining 10.7% of the phenotypic variation (Table 2).

**Table 2:** Associations between SSR and SNP marker loci and Anthracnose (race 4) severity determined by unified mixed linear models (MLM).

Marker	Chromosome (Pv)	p- value^a^	R^2^_ marker^b^	R^2^_ marker (%)
PvM56	1	0.0101*	0.0359	3.59
PvM123	1	0.0235*	0.0244	2.44
PvM15	1	0.0042**	0.0539	5.39
BMc271	1	0.0367*	0.0236	2.36
scaffold00024_916410	1	0.00076488***	0.07697	7.697
PvM93	2	0.0163*	0.0435	4.35
PvM153	2	0.000040712***	0.0877	8.77
PvM126	3	0.002**	0.0661	6.61
SSR-IAC167	3	0.0084**	0.047	4.7
PvM95	3	0.0195*	0.0283	2.83
PvM124	3	0.0482*	0.0209	2.09
PVEST236	3	0.0366*	0.0544	5.44
scaffold00045_345513	3	0.00114**	0.09041	9.041
scaffold00060_874577	4	0.00052705***	0.08115	8.115
scaffold00090_802505	4	0.00121**	0.08733	8.733
PvM07	5	0.031*	0.0502	5.02
scaffold00062_295319	5	0.04492*	0.04494	4.494
PvM14	6	0.0271*	0.0446	4.46
scaffold00001_1947432	6	0.04529*	0.03401	3.401
scaffold00128_112577	6	0.03906*	0.03603	3.603
scaffold00128_197955	6	0.0043**	0.07229	7.229
scaffold00001_2118513	6	0.04286*	0.04503	4.503
scaffold00021_89379	7	0.00058202***	0.10781	10.781
scaffold00088_364454	7	0.01903*	0.05802	5.802
scaffold00021_767280	7	0.03442*	0.03785	3.785
scaffold00094_563857	7	0.01938*	0.04311	4.311
scaffold00098_217812	7	0.0138*	0.03323	3.323
SSR-IAC254	8	0.00097355***	0.0564	5.64
PvM68	8	0.0247*	0.0153	1.53
scaffold00105_48480	8	0.04527*	0.03448	3.448
scaffold00034_860044	8	0.00043065***	0.09668	9.668
scaffold00097_323110	8	0.03266*	0.03752	3.752
scaffold00097_164240	8	0.03657*	0.03631	3.631
PvM98	11	0.00078543***	0.066	6.6
SSR-IAC127	11	0.019*	0.0269	2.69
scaffold00009_1366067	11	0.02796*	0.04973	4.973
scaffold00009_825782	11	0.01282*	0.04752	4.752
scaffold00096_204246	11	0.04505*	0.04573	4.573

^a^ *, p ≤ 0.05; ** p ≤ 0.01; *** p ≤ 0.001.

^b^ R^2^_ marker = The fraction of the total variation explained by the marker after fitting the other model effects.

A total of 11 SSRs (10.6%; Table 3) were associated with ALS (race 0–39) disease, of which two, PvM97 (Pv01) and PvM62 (Pv05), were the most significant *(P* ≤ 0.01; Table 3). A total of 17 associations (5.1%) involving SNPs were associated with ALS disease (Table 3; S2 Fig), and the SNP scaffold00019_566327 (Pv11) showed the highest significance (*P* ≤ 0.01). The marker SSR-IAC66 (Pv04) was most significantly associated with ALS, explaining 16.66% of the phenotypic variation (Table 3).

**Table 3:** Associations between SSR and SNP marker loci and angular leaf spot (race 0–39) severity determined by unified mixed linear models (MLM).

Marker	Chromosome (Pv)	p- value^a^	R^2^_ marker^b^	R^2^_ marker (%)
PvM97	1	0.0041**	0.0407	4.07
SSR-IAC167	3	0.0296*	0.041	4.10
PvM95	3	0.0228*	0.0276	2,76
BMc215	3	0.0447*	0.0218	2.18
SSR-IAC66	4	0.0198*	0.1666	16.66
BMc300	4	0.0169*	0.0327	3.27
BMc225	4	0.0173*	0.0661	6.61
scaffold00060_115096	4	0.0383*	0.03826	3.826
scaffold00060_401853	4	0.04706*	0.04779	4.779
scaffold00076_331846	4	0.04686*	0.02331	2.331
PvM62	5	0.0086**	0.0387	3.87
scaffold00037_358238	6	0.02852*	0.0536	5.36
scaffold00001_2031371	6	0.04272*	0.03811	3.811
scaffold00021_89379	7	0.04195*	0.05666	5.666
scaffold00111_115892	7	0.01048*	0.05333	5.333
scaffold00126_28972	7	0.0329*	0.04073	4.073
scaffold00111_19536	7	0.03029*	0.02764	2.764
PvM01	8	0.0219*	0.115	11.5
scaffold00034_1236020	8	0.02152*	0.04523	4.523
scaffold00041_635678	8	0.04989*	0.04717	4.717
PvM61	9	0.0121*	0.0356	3.56
scaffold00043_193294	9	0.03672*	0.05087	5.087
scaffold00101_378095	9	0.04128*	0.03813	3.813
BMc273	10	0.0197*	0.06	6.00
scaffold00019_1159551	11	0.04345*	0.03716	3.716
scaffold00019_566327	11	0.00194**	0.08497	8.497
scaffold00019_960179	11	0.01919*	0.05845	5.845
scaffold00009_208616	11	0.02388*	0.05398	5.398

^a^ *, p ≤ 0.05

** p ≤ 0.01

***p ≤ 0.001.

^b^ R^2^_ marker = The fraction of the total variation explained by the marker after fitting the other model effects.

The markers SSR-IAC167 and PvM95, both located on chromosome Pv03, and the SNP scaffold00021_89379, were associated with both diseases, suggesting a possible pleiotropic effect.

### SSRs, SNPs, and genes associated with anthracnose and angular leaf spot resistance

**Fig 3:**
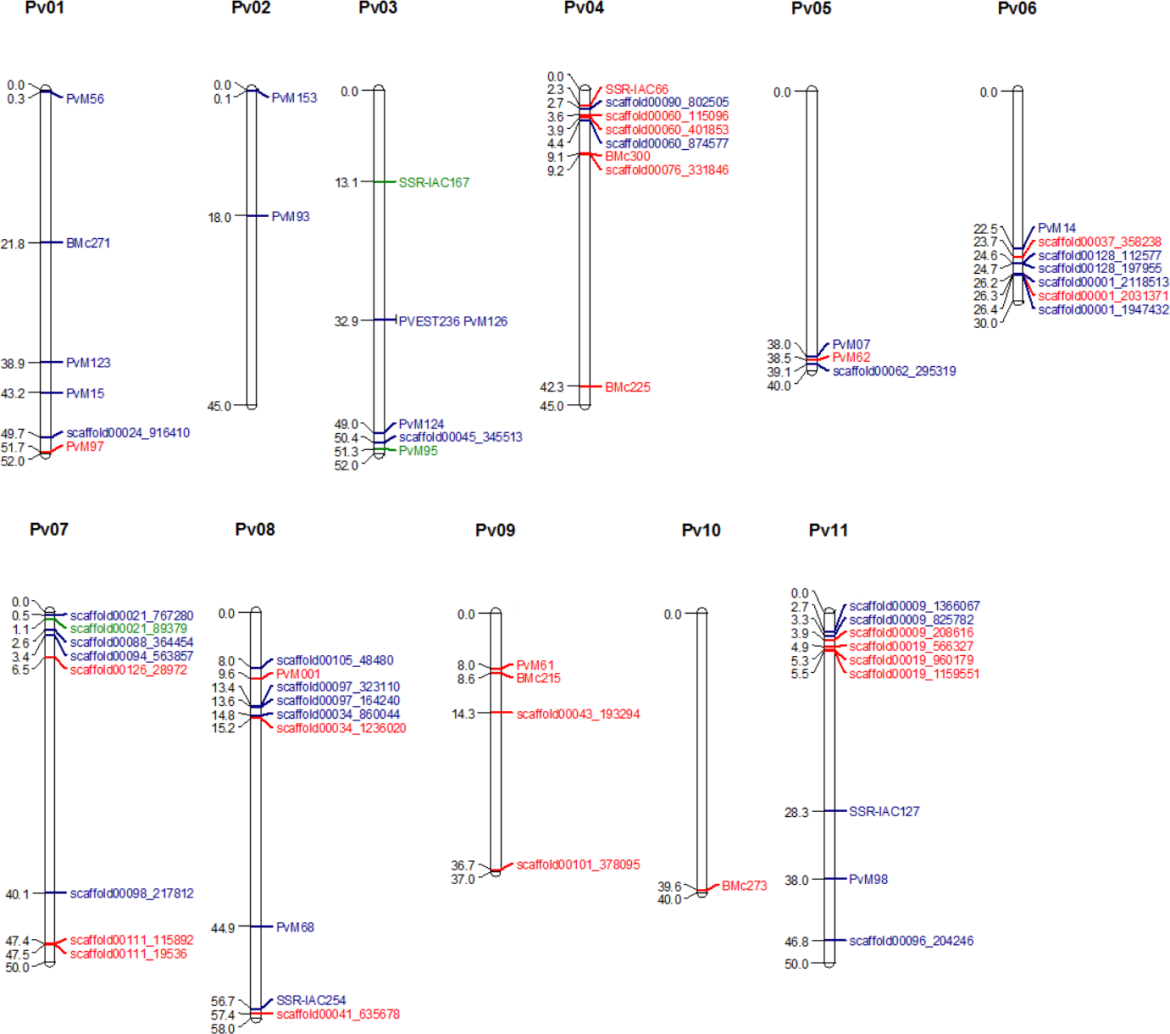
Representative map of the genome positions of markers associated with ANT and ALS resistance. SSR and SNP markers were located on the reference genome (Schmutz et al., 2014) using the Phytozome v1.0 BLAST tool (http://www.phytozome.net/). Markers in blue are associated with ANT resistance and those in red are associated with ALS resistance. The SSR-IAC167, PvM95, and sacaffold00021_89379 markers (green) were associated with resistance to both diseases. The map was drawn with MapChart (Voorrips 2002), and marker positions are represented in Mb.

The SSR and SNP markers found to be associated with both ANT (Table 2) and ALS (Table 3) resistance were physically located on the recently released common bean genome sequence [1]. All 64 markers were mapped to within a certain region on the common bean genome (E-value ≤4 x 10^−50^). In general, the markers were distributed across the entire common bean genome, with chromosomes Pv03 and Pv08 showing the greatest number of loci associated with ANT resistance. The chromosome Pv04 was the most saturated one, with six markers associated to ALS resistance (Tables 2 and 3). The telomeric region of this chromosome showed four markers located between approximately 2.5 Mb and 4.4 Mb (Fig 3).

Taking into account both ALS and ANT, chromosomes Pv03 and Pv11 portrayed the larger number of loci (nine) involved in the immune response to both diseases (Fig 3; S1 and S2 Tables). Telomeres also appeared to be enriched for disease-associated loci in chromosomes Pv06 (22.5 Mb to 26.4 Mb; seven markers), Pv07 (0.5 Mb to 3.4 Mb; four markers), and Pv11 (2.7 Mb to 5.5 Mb; six markers) (Fig 3).

The predicted candidate gene locations in the genome for each marker were identified using the reference common bean genome sequence [1]. A total of five markers were mapped on common bean genome regions with no expressed genes, based on the Phytozome RNA-seq and EST database [1]. In total, 57 genes were identified. Different functions could be assigned to the genes where the markers were located, including five involved with transcription factors, 12 of unknown functions, four kinases, and one with malectin-tyrosine kinase (S1 and S2 Tables).

Gene Ontology (GO) enrichment analysis was performed for all candidate genes to investigate whether the loci associated with ANT and ALS resistance corresponded to genes involved in known pathways. GO accessions, allowed them to be grouped into three functional categories: those related to Biological Processes, Molecular Functions and Cellular Components. Most of SSRs markers associated to Anthracnose were enriched into five biological processes: "Cellular metabolic process", "Biosynthetic process", "Primary metabolic process", "Organic substance metabolic process", "Regulation of biological process", followed by “Nitrogen compound metabolic process” and “Death”, whereas those associated to Angular Leaf Spot were distributed in equal proportions in similar process, except by “Establishment of localization”. In the molecular function and cellular component categories ANT-SSRs showed more categories than ALS-SSRs. Those related to ANT were mostly enriched in following molecular functions: “Sequence–specific DNA binding transcription factor activity”, “Ion binding”, “Heterocyclic compound binding” and “Organic cyclic compound binding”. “Protein binding”, “Oxidoreductase activity”, “Ion binding”, were the main functions assigned to SSR associated to ALS (S3 Fig). “Cell part” and “Membrane-bounded organelle” were the common cellular component for SSRs associated to both diseases.

Regarding to SNPs markers, similarly to SSRs, they were assigned to “Primary metabolic process”, “Organic substance metabolic process”, Cellular metabolic process”, “Establishment of localization”, “Single-organism cellular process” as main biological process for both diseases. In “Molecular Function category”, SNPs were assigned to eleven classes for ANT, with the most frequent corresponding to "Heterocyclic compound binding", "Ion binding", "Organic cyclic compound binding" and "Transferase activity". For ALS-SNPs, they were assigned to six classes, highlighting "Hydrolase activity”. It is interesting to note that only ANT-SNPs were assigned to “Cellular Component”, such as “Membrane part” and “Cell part” and no “Cell component” was found related to ALS-SNPs. In addition, markers linked to genes involved in biological process such as “methylation”, “biosynthetic process” and “programed cell death” are interesting to consider because they it may be involved in roles of stress response (S3 Fig).

**Fig 4:**
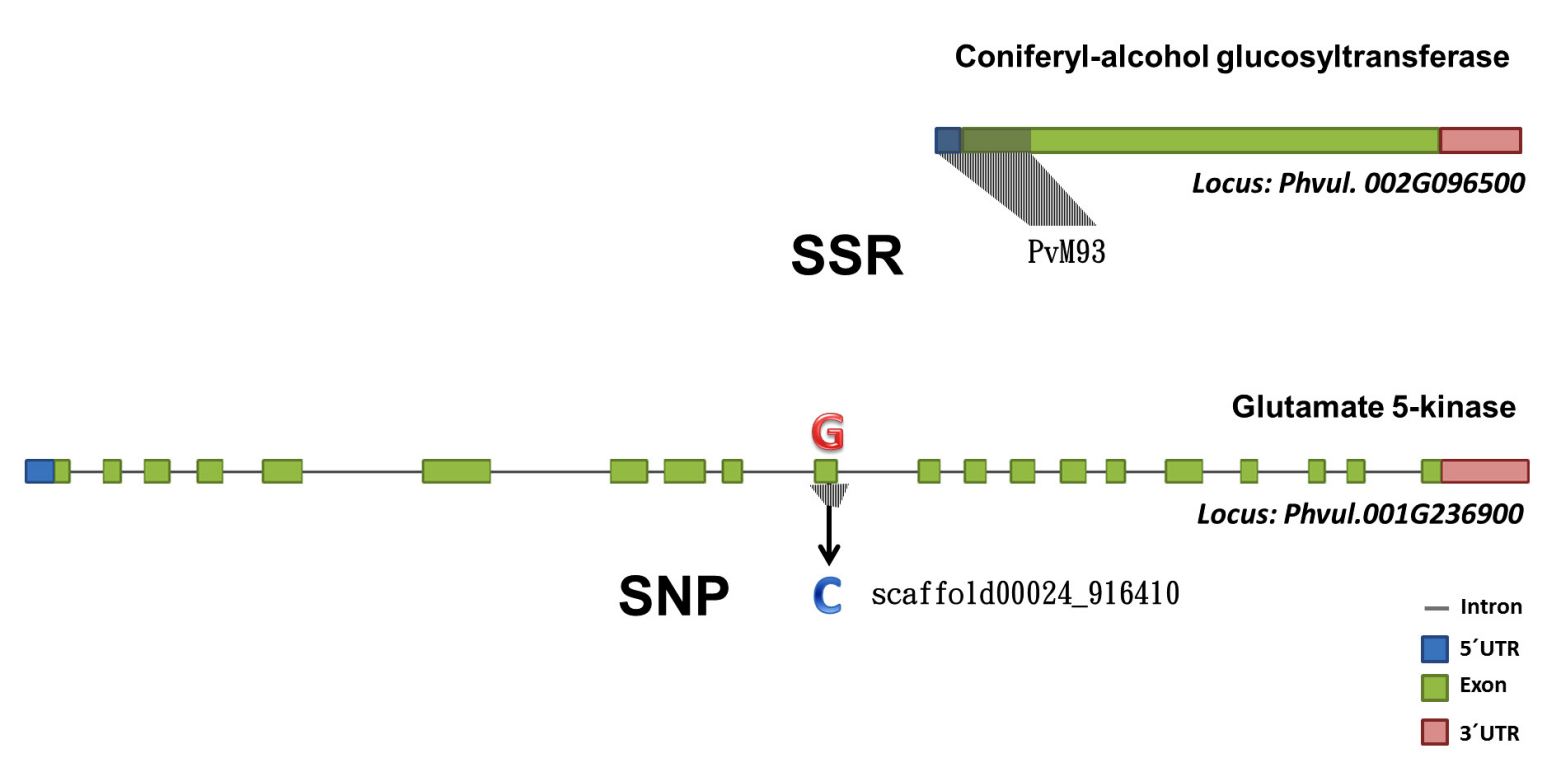
Example of two homologous gene structure predictions showing the marker location in the *locus* PvM93.

Annotation using KEGG allowed the classification of the genes containing SSR and SNPs markers for ANT and ALS in agreement with their function in the context of specific metabolic pathways. Homologous for several enzymes revealed genes involved in the different pathways such as “Phenylpropanoid biosynthesis”, “Porphyrin and Chlorophyll metabolism”, “Carbapenem biosynthesis”, “Aminobenzoate degradation” and “Glycine, Serine and threonine metabolism” (S3 Table). The gene structure prediction allows identify where each marker is located into the gene, what is important to recognize it presence in putative coding regions. For example, specifically the PvM93 marker, which is a SSR associated to ANT, tags a exon of the gene that encode to a Glucosyltransferase involved in “Phenylpropanoid biosynthesis” and the SNP (Scaffold0024_916410) also associated to ANT, tags the gene that encode to a 5-Kinase dehydrogenase involved in “Carbapenem biosynthesis” pathways (Fig 4 and S4 Fig).

## Discussion

The resolution of GWAS depends on the extent and structure of LD detected in the germplasm under consideration. As a predominantly selfing species, extended LD and low rates of effective recombination are expected for common bean [54].

Common bean allows the use of medium-sized populations as diversity panels for AM or GWAS [25,28]. Indeed, the size of the population depends on the relatedness of the individuals, the extent of LD, the type of study, and the polymorphism of the markers. The Brazilian core collection used in this study derived from the Agronomic Institute (IAC, Campinas, SP. Brazil) included domesticated genotypes from Andean and Mesoamerican genepools. A whole-genome-scan association study is feasible for bean domestic populations [21].

Population mating systems could have a strong influence on LD patterns in the common bean. Generally, there is a lower decline of LD in selfing species compared to out-breeding species [55], which could be partially explained by the reduced recombination events or selection effects in selfing species [16]. In this sense, LD tends to remain over tens to hundreds of kilobases in predominantly selfing species, such as rice, soybean, or common bean, showing that population mating could explain the high proportions of LD we detected. Therefore, the large extent of LD observed in this study with SSRs is in accordance with what has been previously reported for the common bean [20,21,22].

The rapid LD decay obtained with SNP markers was not expected. This could be attributed to the fact that these SNPs were developed from sequences that are related to water stress [36] and located within specific regions that are not randomly distributed across the bean genome.

Although LD is generally high in common bean breeding lines, there are some regions where the LD is much reduced, highlighting the importance of estimating LD in the core collection of distributed loci throughout the genome [26].

GWAS is an important tool for gene tagging in crops both for simple traits under additive genetic scenarios, as well as for the dissection of more complex genetic architectures. The advantage of GWAS for QRL discovery over traditional QRL-mapping in biparental crosses is primarily due to (1) the availability of broader genetic variations with wider background for marker-trait correlations (i.e., many alleles evaluated simultaneously), (2) the likelihood of higher resolution mapping because of the utilization of the majority of recombination events from a large number of meioses throughout the germplasm development history, (3) the possibility of exploiting historically measured trait data for associations, and (4) no need for the development of expensive and biparental populations that makes this approach time-saving and cost-effective [26].

According to Ferreira et al., in 2013 20 resistance genes (named *Co*) that condition specific isolates or races of ANT pathogenic on common bean were reported to date [56]. The authors related that the resistance of ANT has been mapped to seven of 11 common bean chromosomes (Pv01; Pv02; Pv03; Pv04; Pv07; Pv08 and Pv11). Recently, Campa et al. in 2014 found new locations for the ANT resistance gene in Pv09 [57].

Most identified ANT resistance genes have been located in the genetic map of common bean: genes *Co*-1, *Co*-x, and *Co*-w were mapped to chromosome Pv01 [58,59,60]; *Co*-u was located to Pv02 [58]; *Co*-13 to Pv03 [61]; *Co*-2 to Pv11 [62]; *Co*-3, *Co*-9, *Co*-y, *Co*-z, and *Co*-10 to Pv04 [59,63,64,65,66,67]; *Co*-4 to Pv08 [59]; and *Co*-5, *Co*-6, and *Co*-v to Pv07 [68,69]. Partial ANT resistance to races 23 and 1545 on Pv5 was previously reported by Gonzalez et al. in 2015 [70]. However, until now, no resistance *locus* to ANT has been found on chromosome Pv06. In fact, the ANT resistance system in common bean has been classically investigated by analyzing a limited number of isolates or races in different segregating populations [57]. Using GWAS, a broader range of genetic variation may be explored, not restricted to a bi parental source. Exploring the genetic architecture of the ANT response in common beans, Oblessuc et al. in 2014 reported a more quantitative response to ANT resistance (race 4), reinforcing the importance of unveiling minor effect alleles that may contribute to durable resistance [32]. Two markers were associated with resistance to ANT on chromosome Pv05 (PvM07 and scaffld00062_295319) and five markers on chromosome Pv06 (PvM14, scaffold00128_112577, scaffold00128_197955, scaffold00001_2118513 and scaffold0001_1947432), revealing new QRLs for ANT resistance.

The PvM07 marker was linked to putative genes related to disease resistance (Zinc finger proteins) (S1 Table). Zinc fingers proteins are members of a super family involved in resistance and regulatory mechanisms for various biotic stresses [71,72]. The presence of zinc finger DNA binding domains in nucleotide binding site-leucine rich repeats (NBS-LRR) determines the regulatory function of this protein in stress conditions [73]. Most plant disease resistance R proteins contain a series of NBS-LRRs. The LRRs of a wide variety of proteins from many organisms serve as protein interaction platforms, and as regulatory modules of protein activation. Genetically, the LRRs of plant R proteins are determinants of response specificity, and their action can lead to plant cell death in the form of the familiar hypersensitive response (HR) [74].

In chromosome Pv02, the PvM13 marker (Fig 3) was co-localized with QRL ANT02.4UC [32]. Three SNPs (scaffold00097_323110, scaffold00097_164240, and scaffold00034_860044; Fig 3) were co-localized with QRL ANT08.2UC [32]. The QRL ANT11.1UC observed in response to ANT race 4 was also positioned on the distal part of chromosome Pv11 [32]. The PvM98 marker was co-localized with this QRL.

Two markers in Pv01 (scaffold00024_916410 and PvM97; Fig 3) were localized in a position corresponding to the *Co*-1 resistance cluster. This resistance cluster was reported recently by Campa et al. in 2014, who found the genes *Co-*1^73-x^ and *Co*-1^65-x^ correspond to the resistance cluster against the ANT races 73 and 65, respectively [57].

Both scaffold00060_115096 and scaffold00060_401853 markers (Fig 3) were associated with ALS on chromosome Pv04. These two markers were localized in a position corresponding to *Co*-10, *Phg*-*ON*, and *Ur*-14 resistance clusters. These three genes appear very important for common bean breeding programs, as indicated by previous studies [63,75].

The ALS resistance was associated with nine of 11 common bean chromosomes (Pv01, Pv02, Pv03, Pv04, Pv05, Pv07, Pv08, Pv09, and Pv10) [76]. Until now, no ALS resistance QRL has been found on chromosome Pv06 (Fig 3). Two markers were associated with resistance to ALS on Pv06: scaffold00037_358238 and scaffold0001_2031371.

Oblessuc et al. in 2012 identified seven QRLs associated with ALS resistance to race 0–39 [30]. Comparing the former results with the associations detected herein, it is possible to verify similar regions for ALS resistance. On Pv03, the PvM124, scaffold00045_345513, and PvM95 markers (Fig 3) were co-localized near the QRL ALS3.1^UC^ [30]. The PvM95 marker was associated with both diseases, while two other markers mapped on this genomic region were assigned specifically to ANT resistance.

On Pv04, BMc225 (Fig 3) was co-localized with QRL ALS4.2^GS, UC^ [30], associated with ALS resistance to race 0–39. Keller et al. in 2015 reported a major QRL on Pv04, around 43.7 Mb, explaining 75.3% of the phenotypic variance to an Andean race (31–0) [76]. BMc225 was located at 42.28 Mb on Pv05, the same genomic region of the Pv-atgc002 marker, linked with ALS4.1^GS,UC^. The QTL5.2UC was co-localized to PvM07, PvM62, and scaffold00062_295319, as they were in the same genomic region of Pv05 that corresponded to this QRL region.

The discovery of gene clusters not only conditioning ANT resistance but linked to other gene clusters conditioning resistance to other pathogens such as ALS, bean rust, bean common mosaic virus, and halo blight has been reported [56]. In our study, new gene clusters were revealed for ANT and ALS resistance, preferentially localized on chromosome telomeres. The end of linkage group B4 was reported to have a high density of resistance genes and RGAs. A major-effect QRL against strain 45 (for leaf, stem, and petiole ANT resistance) and a reverse-effect QRL (i.e., coming from the susceptible Andean JaloEEP558 parent) for leaf ANT resistance against strain A7 were also located in this region [77].

Moreover, functional annotation analyses of markers from homologous sequence provided clues to the molecular bases of putatives responses to stress. In the present study, many GO terms and pathways relevant were identified, like phenylpropanoids, which are a group of plant secondary metabolites derived from phenylalanine, probably having a wide variety of functions as structural and signaling molecules [78]. Phenylalanine is first converted to cinnamic acid by deamination. It is followed by hydroxylation and frequent methylation to generate coumaric acid and other acids with a phenylpropane (C6-C3) unit. Reduction of the CoA-activated carboxyl groups of these acids results in the corresponding aldehydes and alcohols. The alcohols are called monolignols, the starting compounds for biosynthesis of lignin. Obviously, this is important to understanding biochemical bases of regulatory mechanisms induced by hormones in the processes of organogenesis or defense.

Although the genetic basis of durable resistance in plants is not fully understood [79], it is frequently presumed that quantitative resistance, conditioned by “minor” genes and supposed to act in a race-nonspecific manner, would provide durable resistance [80]. As pointed out by Oblessuc et al. positive QRL alleles for ANT and ALS resistance were identified using a recombinant inbred line (RIL) bi-parental population (IAC-UNA x CAL-143) [30,31,32,35]. New associations have been detected in this report using a GWAS approach. GWAS may provide a higher mapping resolution and the possibility to study various regions of the genome simultaneously, since it is based on germplasm collections with minimal genetic structure, preferentially a set of useful accessions for single or multiple traits of interest for breeding programs [54]. The putative genes identified in these regions may be potentially involved in the resistance response. Minor and major effect QRL revealed by this study may play a role in achieving a more comprehensive knowledge of the host-pathogen interactions of *Colletotrichum lindemuthianum* and *Pseudocercospora griseola*. Through cloning and characterizing candidate genes underlying resistance mechanisms in major QRL, the pathways involved in the race-specific and defense response to anthracnose and angular leaf spot infection of common beans can be further elucidated and understood.

## Supporting Information

S1 Fig Linkage disequilibrium (r^2^) of SNP pairs in each of the 11 chromosomes of *Phaseolus vulgaris* L., due to the genetic distance in Mb. (TIF)

S2 Fig Manhattan plots for both diseases. (A) Anthracnose–ANT, (B) Angular leaf spot–ALS. *P* values are shown on a log_10_ scale. Markers are considered significant when *P* ≤ 0.05. Axis x corresponds to the number of chromosomes in common bean.
(TIF)

S3 Fig BLAST2GO annotation of genes containing SSR and SNP markers for Anthracnose and Angular Leaf Spot resistance: Biological Process (BP); Molecular Function (MF); Cellular Component (CC). In parenthesis, there are the numbers of genes of each category.
(TIF)

S4 Fig Phenylpropanoid biosynthesis pathway (map 00940). The blue boxes are for KEGG ECs that have homologies to *Phaseolus vulgaris* sequence target by PvM93.
(TIF)

S1 Table Genomic analysis of molecular markers associated with anthracnose resistance in common bean according to the Phytozome database v1.0. (DOCX)

S2 Table Genomic analysis of molecular markers associated with angular leaf spot resistance in common bean according to the Phytozome database v1.0. (DOCX)

S3 Table Markers that tag with enzymatic functions putatively related to “stress” or “defense”. (DOCX)
