# Supplementary figures and images for: Genome-Wide Association Studies of Anthracnose and Angular Leaf Spot Resistance in Common Bean (Phaseolus vulgaris L.)
Source: PLoS One. 2016 Mar 1;11(3):e0150506. doi: 10.1371/journal.pone.0150506 (PMC4773255; doi:10.1371/journal.pone.0150506)

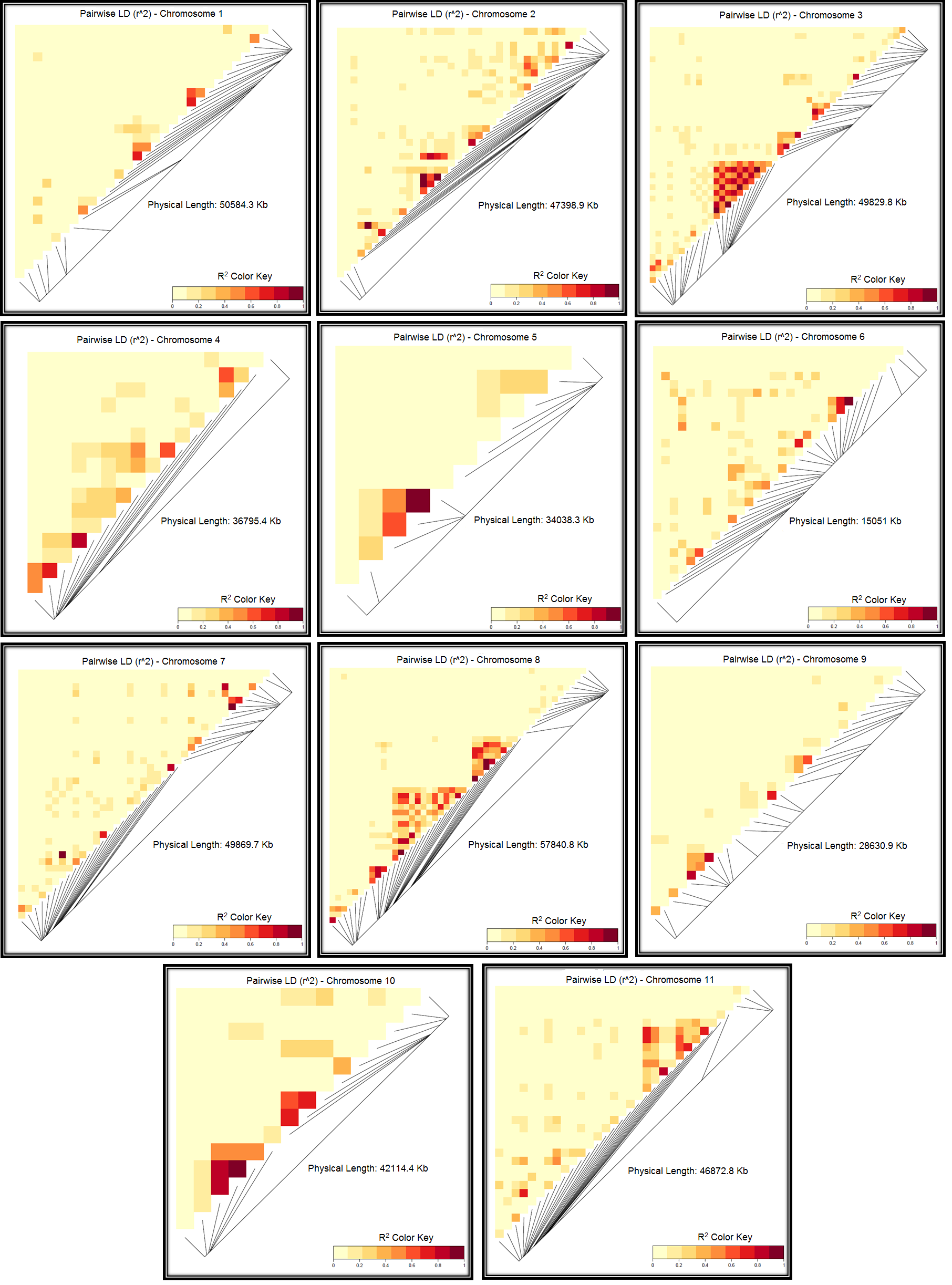

Supplement: S1 Fig — (TIF) [file pone.0150506.s001.tif]

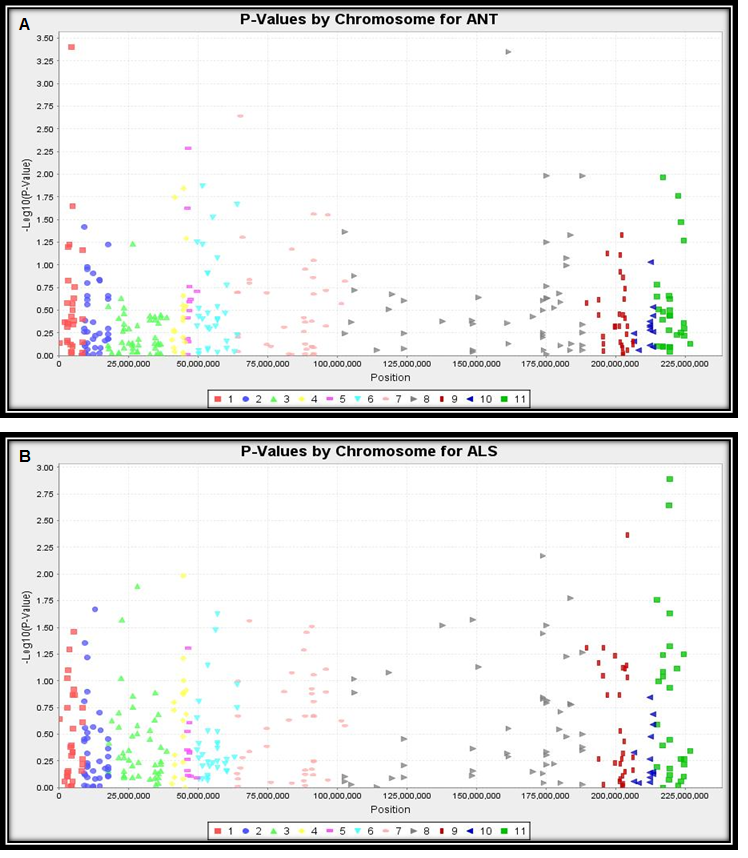

Supplement: S2 Fig — (A) Anthracnose–ANT, (B) Angular leaf spot–ALS. P values are shown on a log10 scale. Markers are considered significant when P ≤ 0.05. Axis x corresponds to the number of chromosomes in common bean. (TIF) [file pone.0150506.s002.tif]

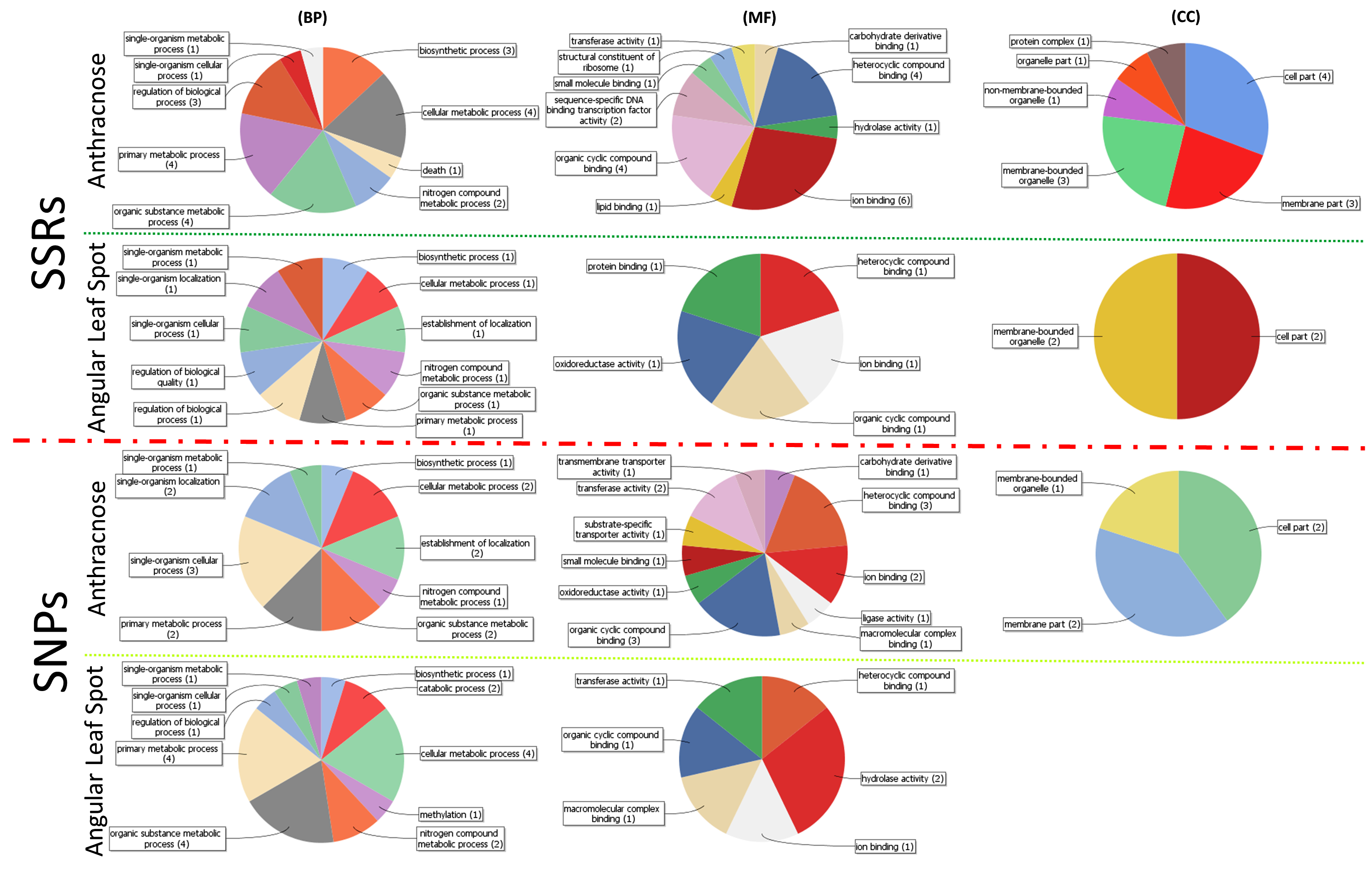

Supplement: S3 Fig — In parenthesis, there are the numbers of genes of each category. (TIF) [file pone.0150506.s003.tif]

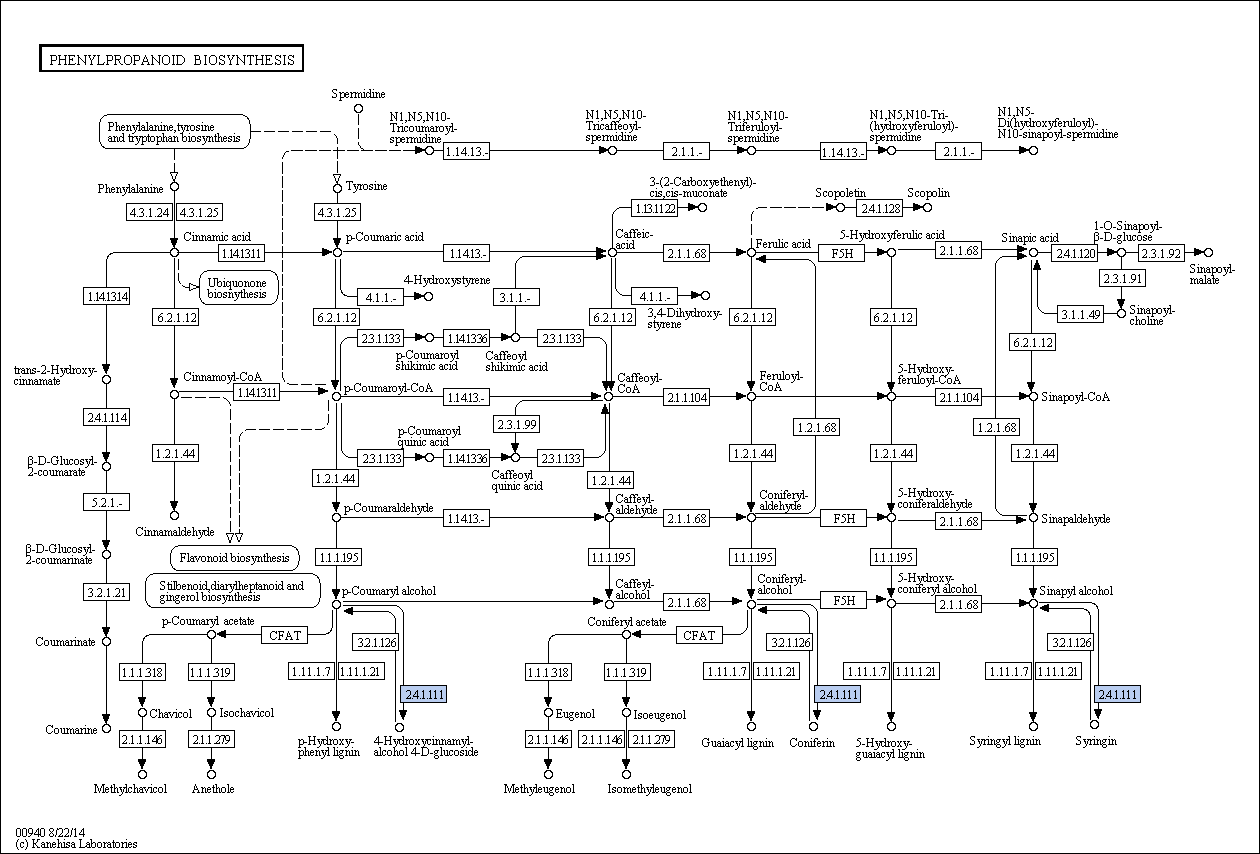

Supplement: S4 Fig — The blue boxes are for KEGG ECs that have homologies to Phaseolus vulgaris sequence target by PvM93. (TIF) [file pone.0150506.s004.tif]
