# Supplementary material for: Genome-Wide Association Studies of Anthracnose and Angular Leaf Spot Resistance in Common Bean (Phaseolus vulgaris L.)
Source: PLoS One. 2016 Mar 1;11(3):e0150506. doi: 10.1371/journal.pone.0150506 (PMC4773255; doi:10.1371/journal.pone.0150506)
Supplement: S1 Table — (DOCX) [file pone.0150506.s005.docx]

**S1 Table**. Genomic analysis of molecular markers associated with anthracnose resistance in common bean according to the Phytozome database v1.0.

| **Marker name** | **Chr^a^** | **E-value** | **Marker genome location (alignment size)** | **Predicted gene** | **Gene genomic location**  **(bp)** | **Functional Annotation^b^** |
| --- | --- | --- | --- | --- | --- | --- |
| PvM56 | 01 | 1 x 10-111 | 260669-260926  (259 b) | Phvul.001G001800 | 260241-260840 | Unknown function |
| BMc271 | 01 | 2 x 10-66 | 21760498-21760654 (158 b) | Phvul.001G098100 | 21757626-21760725 | Glyceraldehyde 3-phosphate dehydrogenase, NAD binding domain |
| PvM123 | 01 | 6 x 10-158 | 38929146-38929864 (720 b) | Phvul.001G141000 | 8925229-38929516 | Squamosa promoter binding (SPB) domain |
| PvM15 | 01 | 0.0 | 43158555-43158931 (378 b) | Phvul.001G168900 | 43158504-43159360 | Unknown function |
| scaffold00024 | 01 | 6 x 10-54 | 49718896-49719017 (123 b) | Phvul.001G236900 | 49717585-49723825 | Aldehyde dehydrogenase and amino acid kinase domains |
| PvM153 | 02 | 2 x 10-118 | 101652-101938  (288 b) | Phvul.002G001400 | 99693-101892 | Inhibitor of apoptosis-promoting Bax1 |
| PvM93 | 02 | 9 x 10-111 | 18030677-18030948 (273 b) | Phvul.002G096500 | 18030456-18032191 | UDP-glucoronosyl and UDP-glucosyl transferase |
| IAC167 | 03 | 0.0 | 13097396-13097848 (454 b) | Phvul.003G081400 | 13096120-13101118 | JmjC domain, hydroxylase |
| PVEST236 | 03 | 0.0 | 32935150-32935964 (816 b) | Phvul.003G137100 | 32935145-32936991 | Transcription factor GATA |
| PvM126 | 03 | 0.0 | 32935183-32935582 (401 b) | Phvul.003G137100 | 32935145-32936991 | GATA zinc finger |
| PvM124 | 03 | 0.0 | 48995346-48995954 (610 b) | Phvul.003G262200 | 48995090-48996005 | Unknown function |
| scaffold00045 | 03 | 3 x 10-51 | 50422102-50422234 (134 b) | Phvul.003G278700 | 50420348-50422308 | ANNEXIN |
| PvM95 | 03 | 1 x 10-57 | 51280966-51281101 (137 b) | Phvul.003G286600 | 51276271-51281707 | Serine Hydroxymethyltransferase |
| scaffold00090 | 04 | 6 x 10-54 | 2701631-2701763 (134 b) | Phvul.004G026500 | 2701204-2703811 | Membrane transport protein |
| scaffold00060 | 04 | 3 x 10-51 | 4395872-4396004 (134 b) | Phvul.004G039600 | 4395440-4398037 | Malectin-tyrosine kinase |
| PvM07 | 05 | 0.0 | 38024011-38024442 (433 b) | Phvul.005G153500 | 38024013-38026445 | B-box zinc finger |
| scaffold00062 | 05 | 6 x 10-54 | 39080673-39080805 (134 b) | Phvul.005G165800 | 39078584-39080893 | PPR repeat |
| PvM14 | 06 | 1 x 10-177 | 22466054-22466490 (438 b) | Phvul.006G107900 | 22466015-22467051 | Late embryogenesis abundant protein |
| scaffold00128_11 | 06 | 1 x 10-49 | 24577146-24577284 (140 b) | Phvul.006G130700 | 24577102-24583854 | Unknown function |
| scaffold00128_19 | 06 | 3 x 10-51 | 24659226-24659358 (134 b) | Phvul.006G131500 | 24658789-24662678 | Unknown function |
| scaffold00001_21 | 06 | 6 x 10-54 | 26202771-26202903 (134 b) | Phvul.006G149300 | 26201595-26206581 | Proteasome inhibitor |
| scaffold00001_19 | 06 | 7 x 10-53 | 26390866-26390998 (134 b) | Phvul.006G150600 | 26389883-26391115 | Heat-shock protein (Hsp20) |
| scaffold00021 | 07 | 7 x 10-53 | 453346-453478  (134 b) | Phvul.007G006200 | 453224-456086 | Heat shock protein binding |
| scaffold00021 | 07 | 7 x 10-53 | 1144040-1144172 (134 b) | Phvul.007G017100 | 1141875-1144255 | FAD binding domain |
| scaffold00088 | 07 | 4 x 10-50 | 2637988-2638118 (132 b) | - | - | - |
| scaffold00094 | 07 | 6 x 10-54 | 3352806-3352938 (134 b) | Phvul.007G041800 | 3345492-3354004 | Transcription factor PHOX2/ARIX |
| scaffold00098 | 07 | 6 x 10-54 | 40094381-40094513 (134 b) | Phvul.007G168500 | 40087589-40122795 | Unknown function |
| scaffold00105 | 08 | 3 x 10-51 | 7988545-7988677 (134 b) | Phvul.008G082900 | 7988247-7991972 | Elongation factor P |
| scaffold00097_32 | 08 | 6 x 10-54 | 13415385-13415517 (134 b | Phvul.008G112600 | 13412167-13417041 | Unknown function |
| scaffold00097_16 | 08 | 6 x 10-54 | 13564031-13564163 (134 b) | Phvul.008G113300 | 13563539-13565503 | Transcription factor, Myb superfamily |
| scaffold00034 | 08 | 6 x 10-54 | 14796002-14796134 (134 b) | Phvul.008G117700 | 14796105-14798123 | Leucine-rich repeat receptor-like protein kinase |
| PvM68 | 08 | 7 x 10-119 | 44860983-44861244 (263 b) | - | - | - |
| IAC254 | 08 | 0.0 | 56681942-56682625 (685 b) | Phvul.008G252600 | 56676409-56683617 | PHOX (PX) domain |
| scaffold00009_13 | 11 | 6 x 10-54 | 2695661-2695793 (134 b) | Phvul.011G031100 | 2693449-2704795 | Alanine--tRNA ligase |
| scaffold00009_82 | 11 | 4 x 10-50 | 3270820-3270951 (133 b) | Phvul.011G037700 | 3270439-3271018 | Auxin responsive protein |
| IAC127 | 11 | 6 x 10-89 | 28334236-28334433 (199 b) | - | - | - |
| PvM98 | 11 | 0.0 | 38007419-38007859 (442 b) | Phvul.011G146900 | 38006130-38007938 | Unknown function |
| scaffold00096_20 | 11 | 6 x 10-54 | 46792860-46792992 (134 b) | Phvul.011G191300 | 46789437-46794291 | Myb transcription factor |

^a^ Chr = chromosome

^b^ Gene functional annotation from Phytozome (http://phytozome.jgi.doe.gov/)
