# Supplementary material for: Genome-Wide Association Studies of Anthracnose and Angular Leaf Spot Resistance in Common Bean (Phaseolus vulgaris L.)
Source: PLoS One. 2016 Mar 1;11(3):e0150506. doi: 10.1371/journal.pone.0150506 (PMC4773255; doi:10.1371/journal.pone.0150506)
Supplement: S2 Table — (DOCX) [file pone.0150506.s006.docx]

**S2 Table.** Genomic analysis of molecular markers associated with angular leaf spot resistance in common bean according to the Phytozome database v1.0.

| **Marker name** | | **Chr^a^** | **E-value** | **Marker genomic location** | **Predicted gene** | **Gene genomic location** | **Functional Annotation^b^** |
| --- | --- | --- | --- | --- | --- | --- | --- |
| PvM97 | 01 | | 0.0 | 51660890-51661335 (447 b) | Phvul.001G262000 | 51661028-51661321 | Unknown function |
| IAC167 | | 03 | 0.0 | 13097396-13097848 (454 b) | Phvul.003G081400 | 13096120-13101118 | JmjC domain, hydroxylase |
| PvM95 | | 03 | 1 x 10-57 | 51280183-51281772 (1.59 Kb) | Phvul.003G286600 | 51276271-51281707 | Serine Hydroxymethyltransferase |
| IAC66 | | 04 | 0.0 | 2333483-2334125  (644 b) | Phvul.004G022800 | 2331418-2334127 | CCR4-NOT transcription complex related |
| scaffold00060_115096 | | 04 | 2 x 10-53 | 3596157-3596288  (133 b) | Phvul.004G032300 | 3590923-3598846 | Calcineurin-like phosphoesterase |
| scaffold00060_401853 | | 04 | 7 x 10-53 | 3926915-3927047  (134 b) | Phvul.004G035400 | 3919544-3927584 | Microtubule binding protein |
| scaffold00076_331846 | | 04 | 7 x 10-53 | 9228241-9228373  (134 b) | Phvul.004G065300 | 9225332-9236079 | Phosphoglycerate kinase |
| BMc300 | | 04 | 4 x 10-34 | 9077669-9077758  (91 b) | Phvul.004G064800 | 9077625-9078545 | Ribulose bisphosphate carboxylase, small chain |
| BMc225 | 04 | | 1 x 10-70 | 42281228-42281428 (202 b) | Phvul.004G143400 | 42276911-42281524 | Myb-like DNA-binding domain |
| PvM62 | | 05 | 7 x 10-174 | 38465017-38465434 (419 b) | Phvul.005G159100 | 38463426-38465441 | Late embryogenesis abundant protein |
| scaffold00037_358238 | 06 | | 6 x 10-54 | 23703612-23703742 (132 b) | Phvul.006G121300 | 23701717-23706599 | Glycosyl hydrolases family 28 |
| scaffold00001_2031371 | | 06 | 6 x 10-54 | 26310626-26310758 (134 b) | Phvul.006G149800 | 26308399-26313804 | Unknown function |
| scaffold00021_89379 | | 07 | 7 x 10-53 | 1144040-1144172  (134 b) | Phvul.007G017100 | 1141875-1144255 | Monooxygenase |
| scaffold00111_115892 | | 07 | 6 x 10-54 | 47440477-47440609 (134 b) | Phvul.007G234000 | 47411861-47442022 | Ubiquitin carboxyl-terminal hydrolase |
| scaffold00111_19536 | | 07 | 6 x 10-54 | 47534617-47534749 (134 b) | Phvul.007G234700 | 47534396-47537571 | Clp amino terminal domain |
| scaffold00126_28972 | | 07 | 6 x 10-53 | 6453012-6453144  (134 b) | Phvul.007G071300 | 6443304-6454806 | RNA polymerase III subunit |
| scaffold00034_1236020 | | 08 | 6 x 10-54 | 15166121-15166253 (134 b) | Phvul.008G119500 | 15165224-15168201 | Haloacid dehalogenase-like hydrolase |
| scaffold00041_635678 | | 08 | 6 x 10-54 | 57396255-57396387 (134 b) | - | - | - |
| PvM001 | | 08 | 0.0 | 9621400-9622943  (1.54 b) | Phvul.008G093700 | 9621458-9624278 | Ferritin-like domain |
| scaffold00043_193294 | | 09 | 6 x 10-54 | 14253108-14253240 (134 b) | - | - | - |
| scaffold00101_378095 | | 09 | 6 x 10-54 | 36679846-36679978 (134 b) | Phvul.009G254000 | 36674885-36680065 | Unknown function |
| PvM61 | | 09 | 0.0 | 8004083-8005066  (985 b) | Phvul.009G039000 | 8001373-8005081 | No apical meristem (NAM) protein |
| BMc215 | 09 | | 4 x 10-36 | 8576305-8576396  (93 b) | Phvul.009G044200 | 8574350-8576730 | NAC and TS-N domains transcription factor |
| BMc273 | 10 | | 1 x 10-41 | 39573581-39573684 (105 b) | Phvul.010G125600 | 39567307-39574835 | Spt20 family |
| scaffold00009_208616 | | 11 | 2 x 10-48 | 3903069-3903201  (134 b) | Phvul.011G045400 | 3902375-3905467 | Unknown function |
| scaffold00019_566327 | | 11 | 7 x 10-53 | 4879741-4879873  (134 b) | Phvul.011G056900 | 4879012-4882844 | Cupin superfamily protein |
| scaffold00019_960179 | 11 | | 6 x 10-54 | 5282166-5282298  (134 b) | Phvul.011G060800 | 5281963-5284111 | Pentatricopeptide (PPR) repeat-containing protein |
| scaffold00019_1159551 | | 11 | 6 x 10-54 | 5480864-5480996  (134 b) | Phvul.011G063200 | 5479679-5481739 | GRAS domain family |

^a^ Chr = chromosome

^b^ Gene functional annotation from Phytozome (http://phytozome.jgi.doe.gov/)
