# Supplementary material for: Genome-Wide Association Studies of Anthracnose and Angular Leaf Spot Resistance in Common Bean (Phaseolus vulgaris L.)
Source: PLoS One. 2016 Mar 1;11(3):e0150506. doi: 10.1371/journal.pone.0150506 (PMC4773255; doi:10.1371/journal.pone.0150506)
Supplement: S3 Table — (DOCX) [file pone.0150506.s007.docx]

**S3 Table.** Markers that tag with enzymatic functions putatively related to “stress” or “defense”.

| **Marker** | **Type** | **Disease** | **Enzyme** | **EC** | **Pathway** | **Map** |
| --- | --- | --- | --- | --- | --- | --- |
| PvM93 | SSR | Anthracnose | Glucosyltransferase | 2.4.1.111 | Phenylpropanoid biosynthesis | 00940 |
| PvM001 | SSR | Angular Leaf Spot | Ceruloplasmin | 1.16.3.1 | Porphyrin and Chlorophyll metabolism | 00860 |
| Scaffold0024_916410 | SNP | Anthracnose | 5-Kinase  dehydrogenase | 2.7.2.11  1.2.1.41 | Carbapenem biosynthesis | 00332 |
| Scaffold0060_115096 | SNP | Angular Leaf Spot | Nitrophenyl phosphatase  phosphatase | 3.1.3.41  3.1.3.2 | Aminobenzoate degradation | 00627 |
| PvM95 | SSR | Anthracnose  And  Angular Leaf Spot | hydroxymethyltransferase | 2.1.2.1 | One carbon pool by folate  Methane metabolism  Glyoxylate and Dicarboxylate metabolism  Glycine, Serine and threonine metabolism  Cyanoamino acid metabolism | 00670  00680  00630  00260  00460 |
